# Acupuncture for Functional Dyspepsia: A Single Blinded, Randomized, Controlled Trial

**DOI:** 10.1155/2015/904926

**Published:** 2015-07-30

**Authors:** Yulian Jin, Qing Zhao, Kehua Zhou, Xianghong Jing, Xiaochun Yu, Jiliang Fang, Zhishun Liu, Bing Zhu

**Affiliations:** ^1^Department of Acupuncture and Moxibustion, Guang An Men Hospital, China Academy of Chinese Medical Sciences, No. 5 Beixiange Street, Xicheng District, Beijing 100053, China; ^2^Department of Radiology, Guang An Men Hospital, China Academy of Chinese Medical Sciences, Beijing 100053, China; ^3^Department of Health Care Studies, Daemen College, Amherst, NY 14226, USA; ^4^Institute of Acupuncture and Moxibustion, China Academy of Chinese Medical Sciences, Beijing 100700, China

## Abstract

In order to investigate the therapeutic potential of acupuncture on patients with functional dyspepsia (FD), patients were randomized to receive acupuncture at classic acupoints with manipulations (treatment group) versus acupuncture at nonacupoints without manipulation (control group) once every other day, three times a week, for one month and were followed up for three months. The primary outcomes included dyspeptic symptoms, quality of life, and mental status. The secondary outcomes included the fasting serum gastrin concentration, and frequency and propagation velocity of gastric slow waves. Sixty patients with FD were included, among whom, four dropped out. After one month's treatment, patients with FD showed significant improvements in primary (in both groups) and secondary (in the eight patients of the treatment group) outcomes as compared with baseline (*P* = 0.0078 to <0.0001); treatment group has better outcomes in all primary outcome measures (*P* < 0.0001 except for SDS (*P* = 0.0005)). Improvements on dyspeptic symptoms persist during follow-up (better in the treatment group). Acupuncture with manual manipulation had better effects on improving dyspeptic symptoms, mental status, and quality of life in patients with FD. These effects may be related to the increased frequency and propagation speed of gastric slow waves and serum gastrin secretion.

## 1. Introduction

Functional dyspepsia (FD) is dyspepsia without evidence of an organic disease that is likely to explain the cause [1]. Based on the Rome III criteria, symptoms of FD may include bloating, belching, early satiety, abdominal distension, nausea, or indigestion during the last three months with symptom onset at least six month ago. These symptoms are categorized into epigastric pain syndrome and postprandial distress syndrome [2]. Pathophysiological factors which may cause FD include genetic predispositions, early family environment, psychosocial factors, abnormal gastric motility, visceral hypersensitivity, inflammation, and bacterial flora [3]. Particularly, gastrointestinal motor abnormalities, altered visceral sensation, and psychosocial factors have all been identified as major pathophysiological changes in FD [3, 4]. The prevalence of FD varies between 11% and 29.2% [5]. In the United States, FD was found in 29.2% of the population, and in the United Kingdom the prevalence was 23.8% [6, 7].

FD greatly decreases patients' quality of life as the symptoms, particularly abdominal pain and indigestion, cause emotional distress, problems with food and drink, and impaired vitality [8]. Patients with FD usually require extensive diagnostic procedures and long-term medical care which in turn place heavy economic burden on patients and the society. Management of FD mainly includes lifestyle modification,* H. Pylori* treatment, acid suppression therapy, prokinetics, antidepressants, and antiflatulents. Despite these treatment options, treatment for FD often remains unsatisfactory [9]. The management of FD is challenging especially when initial drug therapy fails, which is not uncommon [10]. Furthermore, besides side effects, traditional drug therapy has been strikingly shown to have little to no efficacy [11]. For example, benefits from* H. Pylori* treatment were found to be minimal [12]; acid suppression therapy was found to be suboptimal with no apparent effects on dysmotility-like dyspepsia [13].

From a traditional Chinese medicine (TCM) perspective, FD is characterized by disrupted qi flow inside the middle energizer due to external pathogenic factors [14]. The middle energizer refers to spleen and stomach which are responsible for food transformation and transportation. Kidney is responsible for bone health and the generation of bone marrow; excessive physical work consumes kidney energy. Meanwhile, excessive mental work consumes blood and causes imbalance of emotion. Blood is controlled by heart and emotion is regulated by liver. Thus, treatment in TCM including acupuncture should aim to facilitate qi and blood circulation in meridians related to these organs and thus normalizes patient's status of health. With the guidance of these diagnostic and therapeutic principles, therapeutic effectiveness of acupuncture for abdominal pain, abdominal distension, bloating, nausea, and others was well documented in various TCM classics and has been reported in research studies [15–20].

Besides normalization of qi and blood in the affected meridians, modern understandings of these results also lie in pathophysiological research studies, in which researchers found that acupuncture in patients with FD could accelerate solid gastric emptying [17], increase plasma level of neuropeptide Y but not motilin [18], and induce deactivation of the brainstem, anterior cingulate cortex (ACC), insula, thalamus, and hypothalamus in the human body [21]. In addition, acupuncture was also found to enhance normal gastric myoelectrical regularity in both healthy people and patients with diabetic gastric dysrhythmia [22, 23], alters the frequency of gastric slow waves in healthy volunteers [24], and accelerates solid gastric emptying in diabetic gastroparesis [25].

Acupuncture seems to be a promising treatment for FD; however, the aforementioned clinical trials did not investigate the effects of acupuncture on emotional symptoms [15–25], the prevalence of which has been found to be high in patients with FD [26]; placebo effect which is common in both patients with FD and acupuncture procedures will likely add more uncertainties in the therapeutic effectiveness of acupuncture [27, 28], and, finally, not all of the studies performed acupuncture procedures based on TCM principles including the meridian theories, such as an emphasis on Deqi sensations.

In the present study, we aimed to determine (i) the effect of acupuncture on dyspeptic symptoms, quality of life, and mental status in patients with FD; (ii) the effect difference between classic acupuncture based on TCM principles and acupuncture on nonacupoints; and (iii) effects of classic acupuncture on serum gastrin concentration and frequency and propagation velocity of gastric slow waves.

## 2. Material and Methods

### 2.1. Study Design and Setting

This was a single blinded, randomized, controlled trial of manual acupuncture on classic acupoints versus nonclassic acupoints performed at the Department of Acupuncture at Guang An Men Hospital, one of the top teaching hospitals for TCM education in China. Hospital ethics committee approved the study protocol. Participants were recruited through advertisements on local newspapers, posters, and hospital website and signed informed consent before study participation.

An investigator who was not involved in acupuncture procedures and data analyses was responsible for the generation of a random number table, based on which, participants were allocated to receive either classic acupoint (treatment group) or nonclassic acupoint (control group) acupuncture treatments. Participants were blinded to acupuncture procedures.

### 2.2. Participants

For inclusion, patients have to fulfill the Rome III diagnostic criteria for FD. For the last three months with symptom onset at least six months ago, patient has one or more of the following: (1) bothersome postprandial fullness; (2) early satiation; (3) epigastric pain; (4) epigastric burning; and (5) no evidence of structural disease (including at upper endoscopy) that is likely to explain the symptoms. In addition, the following criteria were also met: no mental disorders; would otherwise be healthy; age 18 to 70; nonpregnant; and one week prior to and during participation, cessation of all medication related to the gastrointestinal system, which may include but not be limited to gastric suppression drugs, prokinetics,* H. Pylori *eradication agents, and antidepressants.

Eight patients in the treatment group also signed in for assessment of gastrin concentration and frequency and propagation velocity of gastric slow waves. To measure the differences between patients with FD and healthy adults in these assessments, eight healthy volunteers were also included to match with these eight patients with FD in the present study.

### 2.3. Treatment Protocol

All patients included were randomized into two groups: classic acupoint (treatment) or nonacupoint (control) groups. For the treatment group, acupoints ST36 and KI3 were used in every group members; additional acupoints of GB4, PC6, and HT7 may also be used based on pattern recognition of symptoms. Based on TCM theories, ST36 was used to invigorate functions of the stomach and spleen; KI3, the Yuan-source acupoint, was used to invigorate functions of the kidney. ST36 and KI3 function together to restore the normal qi flow inside the stomach and spleen meridian. In addition, for patients with obvious depression, anxiety, or insomnia symptoms, GB41 was used to restore liver function, and PC6 and HT7 were used to nourish the heart to resume balance of the mind. Classic acupoints were localized according to the 2008 World Health Organization standards [29]. For the treatment group, needle insertion was perpendicular with a depth of about 25 mm. In order to reach an optimal response which is defined as Deqi sensations including soreness, heaviness, fullness, propagation of needling sensation, and/or adjacent muscle twitching [30], moderate combined acupuncture manipulation of lifting, thrusting, and twirling with a frequency of 60–120 times/min was performed. These acupuncture manipulation techniques were performed continually to reach one to three times of Deqi sensation (with a short interval between Deqi sensations if more than once during the first two minutes); then, the needle was removed. If no Deqi sensation was obtained during the first two minutes, acupuncture needle was then left in place for 20 to 60 minutes, and one acupuncture manipulation was applied right before needle removal regardless of Deqi sensation.

For the control group, nonclassic acupoints in different dermatomes but close proximity of the aforementioned acupoints were used in the distal portion of extremities correspondingly. KI3, ST36, and GB41 are located in the L4, 5, and S1 dermatome; thus nonclassic acupoints located inside anterior thigh (L2 and L3 dermatome) were used. PC6 and HT7 are located inside the C7, 8, and T1 dermatome; thus nonclassic acupoint in the anterior antebrachium (C5 dermatome) was used. In the control group, needle insertion was perpendicular with a depth of two to three millimeters with needle retention of 20 minutes but no acupuncture manipulations.

Treatments in both groups were implemented once every other day, three to four times a week for one month. All patients were then followed up for three months. All acupuncture procedures were performed by the same acupuncturist who had more than six years' clinical experiences. Huatuo brand needles (Φ 0.35 mm × 25 mm, manufactured by Suzhou Medical Appliance manufactory, Jiangsu, China) were used for all acupuncture procedures.

### 2.4. Outcome Assessment

The primary outcomes of the study included dyspeptic symptoms, quality of life, and mental status. For dyspeptic symptoms, we used the four cardinal dyspeptic symptoms and their corresponding assessments as reported in the Chinese version Nepean Dyspepsia Index (NDI) [31, 32]. The intensity, frequency, and level of interference of postprandial fullness, early satiety, epigastric pain, and epigastric burning sensation were rated. Intensity of each symptom was graded and scored as the following: 0, absent; 1, mild; 2, moderate; 3, severe; 4, critical. Frequency of each symptom was also graded as follows: 0, absent; 1, occasionally (1-2 days/week); 2, sometimes (3–5 days/week); 3, frequently (every day, but intermittent symptoms), 4, continuous symptoms. Level of interference of each symptom was scored and graded as the following: 0, none; 1, mild interference; 2, moderate interference; 3, severe interference; 4, critic interference. The number in front of each grading indicates the score of the corresponding symptom; the score for each symptom in the checklist of cardinal dyspeptic symptoms was calculated by adding its scores in the corresponding frequency, severity, and level of discomfort; dyspeptic symptom sum score (DSSS) is the sum score of the four symptoms in the checklist.

Quality of life was measured by the short-form 36 (SF-36) questionnaires [33]. Mental statuses of patients were evaluated via Zung Self-Rating Depression Scale (SDS) [34] and Self-Rating Anxiety Scale (SAS) [35]. Scoring of these standardized assessments followed guidelines published in the Manual of Standardized Assessment Tools in Behavioral Medicine [36]. SF-36 measures Quality of Life (QoL) across eight domains; score of each domain = [(actual raw score − lowest possible raw score)/raw score range] × 100. For the SDS score, the following equation was used: SDS Index = Raw Score × 1.25. Grading of SDS is as the following: SDS Index less than 53 points is considered normal, 53 to 62 as mild depression, 63 to 72 as moderate depression, and 73 and higher as severe depression [36]. For the grading of SAS, the following categories were used: normal range (less than 50), mild anxiety (50 to 59), moderate anxiety (60 to 69), and severe anxiety (70 and higher) [36].

The secondary outcomes include fasting serum gastrin concentration and frequency and propagation velocity of gastric slow waves. These measurements were performed in the eight patients with FD in the treatment group before and after treatment, but only once in healthy volunteers. A fasting venous blood sample was drawn from the basilic vein prior to breakfast early in the morning. About three milliliters of the blood sample was sent to Peking Union Medical College Hospital for measurement of serum gastrin levels. Meanwhile, the participant was given 120 mL 80% (w/v) barium sulfate suspension (Qingdao Dongfeng Chemical Co. Ltd., Shandong, China). Participants were then placed in a supine position. Using Prestige digital X ray (GE, USA), gastric mucosa was observed; then, a Chinese coin of fifty cents was placed on top of the skin over the stomach of the participant, and gastric motions around the gastric antrum were video recorded for one minute while the participant was in a standing position. Frequency of gastric slow waves was directly counted as the number of waves that passed through the gastric antrum in one minute. Propagation velocity of gastric slow waves was assessed by the time interval between two consecutive waves that passed through the gastric antrum.

Safety evaluation includes possible hematoma, local infection, fainting, and severe pain during and after acupuncture. In addition, other conditions which warrant cessation of acupuncture treatment or withdrawal from the study if any were also documented and analyzed.

### 2.5. Statistical Analysis

The statistical analysis was performed by two independent statisticians. Results were compared between the two statisticians. Differences, if any, were discussed and the statistic test was reperformed until a consensus was reached between the two statisticians. The statisticians were blinded to treatments and study protocol. All results including baseline characteristics were based on per-protocol (PP) analyses. Statistical Analysis System (SAS), version 6.12, was used and a significance level was set at *P* < 0.05.

For comparisons of baseline values, chi square test was used to explore gender differences; *t*-test was used to explore differences in the duration of the disease; Wilcoxon rank sum test was used in all comparisons of primary and secondary outcome measures. All quantitative data including subjective scores were expressed with mean ± SD.

## 3. Results

**Figure 1:**
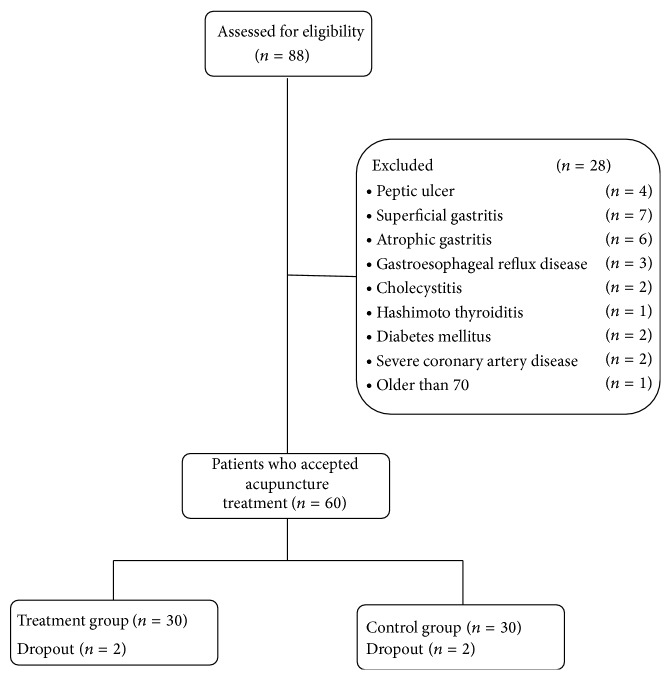
Flow chart of study participation.

From July, 2010, to January, 2011, a total of 88 patients with dyspeptic symptoms visited the Department of Acupuncture at Guang An Men Hospital in Beijing. Twenty-eight patients were excluded from the present study due to the following reasons: peptic ulcer (four patients), superficial gastritis (seven patients), atrophic gastritis (six patients), gastroesophageal reflux disease (three patients), cholecystitis (two patients), Hashimoto thyroiditis (one patient), diabetes mellitus (two patients), severe coronary artery disease (two patients), and older than 70 (one patient). Sixty patients were included and randomly assigned to either the treatment group or the control group. Of these 60 patients, 56 patients completed the study and four patients (two from each group) dropped out from the study (dropout rate: 6.67%) after the second visit. In the treatment group, one patient could not tolerate the acupuncture Deqi sensations upon needle manipulation, and the other patient in the treatment group had transportation difficulties. In the control group, the two patients directly stated to the therapist saying that the treatment was noneffective and withdrew from participation (Figure 1).

The treatment group consists of 11 males and 17 females with an age range between 23 and 65 years old and disease history of one to 40 years. The control group consists of 10 males and 18 females with an age range between 24 and 66 years old and disease history of one to 40 years. Prior to participation, no significant differences were found between these two groups in terms of gender, age, length of disease history, dyspeptic symptom sum scores, and SF-36 score (Table 1).

### 3.1. Primary Outcomes

At baseline, the prevalence of the four symptoms of postprandial fullness, early satiety, epigastric pain, and epigastric burning sensation in these 56 patients were 98.2%, 71.4%, 76.8%, and 58.9%, respectively; the scores for each symptom were six to nine points with a severity of disease rated moderate to severe.

After one month's treatment, as compared with baseline values, significant differences were found in both treatment and control groups in the dyspeptic symptom sum score, the scores of postprandial fullness, early satiety and epigastric pain, SDS score, and SF-36 score. Additionally, as compared with baseline, significant differences were also found in the score of epigastric burning sensation and SAS score of the treatment group but not the control group. *P* values were <0.0001 for all the significant intragroup comparisons except epigastric pain, SF-36, and SDS in the control group, for which *P* values were 0.0078, 0.0099, and 0.0002, respectively. As compared with the control group, treatment group has better outcomes in all primary outcome measures. *P* values for these intergroup comparisons were all <0.0001 except for SDS (*P* = 0.0005) (Table 2).

At three months' follow-up, DSSS was recalculated for all participants. As compared with baseline values, significant differences were found in both groups in terms of DSSS (*P* < 0.0001). Meanwhile, the treatment group, as compared with the control group, had better long-term outcomes in terms of DSSS (*P* < 0.0001) (Table 3).

### 3.2. Secondary Outcomes

Values of preprandial serum gastrin concentration and frequency and propagation velocity of gastric slow waves in healthy volunteers and patients with FD were provided in Table 4. As compared with healthy volunteers, patients with FD had lower serum gastrin concentration and less frequent and slower propagation velocity of gastric slow waves (*P* = 0.0081, 0.0008, 0.0279, resp.) at baseline. After one month's treatment, patients with FD showed significant improvement in serum gastrin concentration and frequency and propagation velocity of gastric slow waves (*P* = 0.0002, 0.0078, and 0.0180, resp.), and no significant difference was found in these secondary outcome measures between healthy volunteers and patients with FD (Table 5).

### 3.3. Side Effects

No serious side effects occurred. One patient in the treatment group withdrew from the study secondarily to intolerance to the needling sensations upon acupuncture manipulation.

## 4. Discussion

### 4.1. Selection of Acupoints

The use of classic acupoint of ST36 in the present study is well-supported by former research studies [15–25], so was the use of PC6 [15–19, 24]. In previous research studies, researchers mainly considered the pathophysiological relationship between the meridians or organs of liver and spleen, heart and spleen, or spleen and kidney; acupoint of the kidney meridian is barely used for FD in these research studies [15–25]. In the present study, we used KI3 based on the analysis of all the pathophysiological relationships between and among organs and meridians related to FD symptoms. These diagnostic and therapeutic principles would be a more realistic reflection of individualized acupuncture treatment in clinical practice. The results of the present study add further credence to the use of these acupoints.

### 4.2. Acupuncture Manipulations

Acupuncture Deqi serves as the foundation or premise for the therapeutic effects of acupuncture treatment [30]. Although theoretical research articles highlight the importance and the components of Deqi, not many researchers emphasized Deqi in their reports of acupuncture clinical trials. The reason for lacking of information regarding Deqi may be due to the following reasons: the authors of the reports did not document it and the clinicians did not pay extra attention to the importance of Deqi during the studies. In addition, as electroacupuncture becomes more and more popular, the evaluation of Deqi is more difficult due to the mixture of electric therapy sensation with sensations from acupuncture itself. Nonetheless, report of Deqi in clinical trials reflects more the standard acupuncture treatment in clinical practice. In the present study, manual acupuncture manipulation was stopped and the needle was removed upon Deqi arrivals in the treatment group. This acupuncture treatment protocol guarantees not only the Deqi sensations thus the clinical efficacy but also safety of acupuncture treatments. The results of the present study indicate that traditional acupuncture with the emphasis of Deqi manipulations has better therapeutic results than acupuncture on nonacupoints without Deqi manipulations.

### 4.3. Outcome Measurement

As psychosocial factor is a common cause of FD and many patients of FD have anxiety or depression issues, measurements of these psychological symptoms are of great importance in the evaluation of clinical management of FD [1, 26]. Zung Self-Rating Depression Scale (SDS) and Self-Rating Anxiety Scale (SAS) have a high reliability and validity in assessing psychological symptoms in patients [34, 35]. The improvement of SDS score and SAS score in the treatment group of the present study indicates that acupuncture has positive impacts on the psychological aspects of patients with FD. Psychological effects of acupuncture may be caused by placebo effects [28, 37]; however, as acupuncture also demonstrated therapeutic effects on psychological diseases [38, 39], we should increase our trust on the positive benefits of acupuncture on psychological symptoms of patients. To our best knowledge, no studies have explored the effects of acupuncture on psychological symptoms of FD. Thus, the present study will facilitate our understanding of the therapeutic effectiveness of acupuncture in FD.

Quality of life is a heavy emphasis of the clinical management of all kinds of disorders. In the present study, the use of modified NDI is well-supported by its high reliability and validity in patients with dyspeptic symptoms [31, 32]. NDI measures dyspepsia symptoms and dyspepsia-specific health-related QOL (H-QOL). Outcome measurements utilizing NDI, SAS, and SDS will likely better capture the characteristics of acupuncture effects on FD. The improvement of NDI in the present study concurs with results from other acupuncture researchers regarding acupuncture treatment for FD [15–21]. Interestingly, the control group in which acupuncture was used in nonclassic acupoints also induced significant changes in dyspeptic symptoms except for epigastric burning sensations and SAS score. These results partially concur with the results reported by Ma et al. [20] and Zeng et al. [21]; however, the results differ from the results reported by Park et al. [19]. Significant superiority of classic acupoint acupuncture to nonclassic acupoint acupuncture was found in both studies by Ma et al. [20] and Zeng et al. [21]; however, they did not report changes of subcategories of NDI in both groups which makes the analysis difficult. Park et al. [19] did not find difference between classic acupoint acupuncture and nonclassic acupoint acupuncture except for pressure and cramps in upper abdomen (better results in the classic acupoint acupuncture group). The differences may be due to control group treatment. In the study by Park et al. [19], dermatome information between classic acupoint and nonclassic acupoint was not included in consideration upon the design of control group.

FD, like other diseases, is characterized by its objective physiological changes and subjective symptoms; thus, a thorough evaluation of FD should simultaneously include these two aspects. Changes in gastric motility, mainly gastric hypomotility or dysrhythmias, play an important role in the pathophysiology of FD [1–4, 13]; thus, an objective measurement of gastric motilities is of great importance in the evaluation of FD. Barium sulfate radiography of the GI system provides a direct visual observation of the frequency and propagation velocity of gastric slow waves and thus objective measurements of acupuncture effects on FD. Gastrin is a peptide hormone that stimulates the secretion of gastric acid by the parietal cells of the stomach and aids in gastric motility. Results from recent research studies indicate that abnormal gastrin level is a possible contribution factor of FD with gastric dysmotility [4, 40, 41]. The results of gastrin level in the present study are consistent with the hypothesis as patients with FD show decreased preprandial gastrin level [4, 41]. However, He et al. [40] did not find any difference in preprandial but postprandial gastrin levels (higher in patients with FD) between patients with FD and healthy volunteers. In the present study, observation of less frequent and slower propagation velocity of gastric slow waves in patients with FD via barium sulfate radiography also indicates a decreased level of gastrin. Nonetheless, changes of gastrin and gastric motility in patients with FD deserve further research. In the present study, classic acupuncture was found to increase preprandial gastrin level and enhance gastric motility of patients with FD to reach similar levels as healthy volunteers. These results are consistent with our findings in improvement of dyspeptic symptoms.

### 4.4. Therapeutic Mechanism of Acupuncture

Former research studies in human beings indicate that acupuncture could accelerate solid gastric emptying [18, 25] and enhance percentage of normal gastric slow waves [22, 23]. The present study showed similar results in increasing gastric motility as demonstrated by increased frequency and propagation velocity of gastric slow waves in patients with FD.

An accepted mechanism of acupuncture on the functions of the gastric system is related to its effects on the autonomic nervous systems, which Takahashi [15] summarized as follows: acupuncture at the lower limbs (ST36) causes gastric muscle contraction via stimulating the somatoparasympathetic pathway whereas acupuncture at the upper abdomen causes gastric muscle relaxation via stimulating the somatosympathetic pathway. As both main acupoints KI3 and ST36 used in the present study are located in the lower extremities, the result of enhanced gastric motility is likely to be caused by activation of the somatoparasympathetic pathway increasing the secretion of gastrin and other hormones.

Furthermore, acupuncture has also been found to induce changes in cerebral cortex activities of patients with FD [21]. Consequently, we hypothesize that effect of acupuncture on the gastrointestinal system is related to its effects on the peripheral nervous system, central nervous system, and the endocrine systems related to the GI tract. However, to prove the specific causal relationship among these systems, further research studies are needed.

## 5. Limitations

As blinding is difficult in acupuncture studies, the establishment of a blank control group seems impossible. Although nonclassic acupoint acupuncture procedures were used as control in the present study, they are still acupuncture procedures; thus we could not rule out the cofounding factor of needling and placebo effects in the present study. This study is performed at one clinical center with one acupuncturist on a relatively small sample; the results of the present study may not well characterize the response of patients with FD to acupuncture treatments. In addition, the analysis of the results did not include patients who dropped out; data processing based on per protocol population may decrease the credence of the results. To better capture the response of patients with FD to acupuncture, further large scale, multicenter, randomized placebo controlled trials are warranted.

## 6. Conclusion

Classic acupuncture with manual manipulation could improve dyspeptic symptoms, mental status, and quality of life in patients with FD and is superior to nonclassic acupoint acupuncture without manipulations. These effects may be related to the increased frequency and propagation speed of gastric slow waves as well as increased serum gastrin secretion.

## Figures and Tables

**Table 1:** General characteristics of patients with FD prior to participation.

Groups	Cases (*n*)	Gender		Age (year)	Disease duration (years)	Dyspeptic symptom sum score	SF-36 score
		Male (*n*)	Female (*n*)				
Treatment	28	11	17	49.29 ± 10.32	12.20 ± 12.20	24.32 ± 8.28	52.51 ± 13.94
Control	28	10	18	48.25 ± 11.40	12.11 ± 10.20	24.79 ± 7.48	54.06 ± 16.41
*P* value		0.7825		0.7229	0.6145	0.8265	0.7043

**Table 2:** Scores of dyspeptic symptoms, quality of life, and mental status before and after the treatment.

Items	Groups	*N*	Baseline	After treatment	Difference	Improvement rate	*P* value
PF	Treatment	28	9.00 ± 2.09	1.57 ± 2.28	7.43 ± 2.47	82.56%	<0.0001
	Control	27	8.89 ± 2.39	6.22 ± 2.59	2.67 ± 1.88	30.03%	<0.0001
	IGC						<0.0001

ES	Treatment	19	9.74 ± 1.91	0.42 ± 1.43	9.32 ± 1.97	95.69%	<0.0001
	Control	21	8.43 ± 2.87	6.05 ± 2.52	2.38 ± 1.80	28.23%	<0.0001
	IGC						<0.0001

EP	Treatment	21	6.81 ± 2.23	0.48 ± 1.03	6.33 ± 2.31	92.95%	<0.0001
	Control	22	7.41 ± 3.02	6.32 ± 3.41	1.09 ± 1.82	14.71%	0.0078
	IGC						<0.0001

EBS	Treatment	16	6.31 ± 2.39	0.50 ± 1.55	5.81 ± 2.17	92.08%	<0.0001
	Control	17	6.71 ± 2.78	6.47 ± 3.00	0.24 ± 0.56	3.58%	0.25
	IGC						<0.0001

DSSS	Treatment	28	24.32 ± 8.28	2.50 ± 3.28	21.80 ± 8.24	89.72%	<0.0001
	Control	28	24.79 ± 7.48	19.40 ± 8.23	5.36 ± 3.29	21.62%	<0.0001
	IGC						<0.0001

SF-36	Treatment	28	52.50 ± 13.94	70.00 ± 12.54	17.00 ± 14.04	33.52%	<0.0001
	Control	28	54.00 ± 16.41	56.00 ± 13.42	2.88 ± 8.74	5.33%	0.0099
	IGC						<0.0001

SDS	Treatment	28	57.96 ± 9.55	45.60 ± 8.75	12.30 ± 9.89	21.33%	<0.0001
	Control	28	57.60 ± 11.84	54.00 ± 10.80	3.50 ± 5.92	6.07%	0.0002
	IGC						0.0005

SAS	Treatment	28	52.30 ± 10.48	42.30 ± 6.22	10.00 ± 10.22	19.11%	<0.0001
	Control	28	52.36 ± 9.67	52.20 ± 7.98	0.11 ± 4.89	0.21%	0.8533
	IGC						<0.0001

PF: postprandial discomfort; ES: early satiety; EP: epigastric pain; EBS: epigastric burning sensation; DSSS: dyspeptic symptom sum score; SF-36: short-form 36 questionnaire; SDS: Self-Rating Depression Scale; SAS: Self-Rating Anxiety Scale; IGC: intergroup comparison.

**Table 3:** Dyspeptic symptom sum score at baseline and during follow-up.

IND	Groups	*N*	Baseline	Follow-up	Difference	Improvement	*P* value
DSSS	Treatment	28	24.32 ± 8.28	1.68 ± 2.36	22.60 ± 8.68	93.09%	<0.0001
	Control	28	24.79 ± 7.48	16.43 ± 7.41	8.36 ± 6.58	33.92%	<0.0001
	IGC						<0.0001

DSSS: dyspeptic symptom sum score; IGC: intergroup comparison.

**Table 4:** Serum gastrin concentration and frequency and propagation velocity of gastric slow waves in patients with functional dyspepsia and healthy adults.

Items	Baseline (*n* = 8)	After treatment (*n* = 8)	Healthy adults (*n* = 10)
Gastrin (pg/mL)	25.93 ± 5.90	44.40 ± 6.26	47.65 ± 20.21
FGSW (n/min)	2.49 ± 0.64	3.11 ± 0.14	3.11 ± 0.13
PVGSW (s)	24.25 ± 4.95	19.75 ± 2.05	19.41 ± 0.93

FGSW: frequency of gastric slow waves; PVGSW: propagation velocity of gastric slow waves.

Note: propagation velocity of gastric slow waves was assessed by the time interval between two consecutive waves that passed through the gastric antrum.

**Table 5:** Comparisons of serum gastrin concentration and frequency and propagation velocity of gastric slow waves before and after treatment as well as between patients with functional dyspepsia and healthy adults.

Items	Baseline versus healthy adults	Baseline versus after treatment	After treatment versus healthy adults
Gastrin	0.0081	0.0002	0.6401
FGSW	0.0008	0.0078	1.0000
PVGSW	0.0279	0.0180	0.6713

FGSW: frequency of gastric slow waves; PVGSW: propagation velocity of gastric slow waves.

Note: propagation velocity of gastric slow waves was assessed by the time interval between two consecutive waves that passed through the gastric antrum. “Baseline versus after treatment” refers to patients with functional dyspepsia only.
